# Optimizing Permanganic Acid Production: Effects of Temperature on Stability

**DOI:** 10.3390/mps8060131

**Published:** 2025-11-02

**Authors:** Abdel Elfatah Bakhite Adam, Tomo Suzuki-Muresan, Aditya Rivonkar, Marcel Mokili

**Affiliations:** 1 SUBATECH Laboratory (IMT Atlantique, CNRS/IN2P3, Université de Nantes), 44307 Nantes, France; 2 Mokili Consulting, 44300 Nantes, France

**Keywords:** permanganic acid production, ion exchange resin, potassium permanganate, thermal stability, chromium oxide dissolution, nuclear reactor decontamination

## Abstract

In the nuclear industry, the decontamination of nuclear metallic structures is an essential process to reduce radiation exposure during maintenance or dismantling. The oxide layer, such as chromium (III) oxide (Cr_2_O_3_), formed on stainless steel and nickel-based alloys, contributes significantly to surface radioactivity by trapping radioactive contaminants. To address this, permanganic acid (HMnO_4_) has proven to be a promising oxidizing agent for dissolving these oxide layers—particularly chromium oxide—on stainless steel and nickel-based alloys. In this study, HMnO_4_ was synthesized via ion exchange using AmberLite IRN97 H resin and potassium permanganate (KMnO_4_). The optimized process yielded a highly acidic solution (pH~1.6) with potassium concentrations below 0.1 ppm, indicating near-complete exchange efficiency. Dissolution kinetics were investigated at HMnO_4_ concentrations ranging from 240 to 1920 ppm and temperatures from 30 °C to 80 °C. At a constant temperature, increasing HMnO_4_ concentration significantly improved Cr dissolution, with up to 31% of total chromium solubilized after 33 h. Lower temperatures favored higher dissolution efficiency, likely due to improved thermal stability of HMnO_4_. For durations shorter than 4 h, the influence of temperature was limited compared to the effect of acid concentration. To assess post-treatment options, HMnO_4_ decomposition was studied using oxalic acid (H_2_C_2_O_4_) at 80 °C. Results showed that a minimum H_2_C_2_O_4_/HMnO_4_ molar ratio above 2.75 was necessary to achieve effective reduction while preventing MnO_2_ precipitation. However, even under strongly acidic conditions and with a large excess of reductant, Mn^2+^ yields remained below 55%, suggesting that thermal degradation of oxalic acid and possible formation of undetected manganese species limited the reduction process.

## 1. Introduction

Permanganic acid is a powerful oxidizing agent derived from the permanganate anion Mn$O_{4}^{-}\;$which has a standard redox potential E^0^ (Mn$O_{4}^{-}$/Mn^2+^) = 1.507 V [1], a monovalent species known for its vibrant purple coloration in aqueous solution, and its solid dihydrate from HMnO_4_·2H_2_O [2]. Permanganic acid is a strong acid with several pKa values proposed in the literature. The values vary between −4.5 and −2.25 [3,4,5]. Understanding the production mechanisms and optimizing the synthesis of permanganic acid HMnO_4_ are essential to enhance its range of applications while mitigating safety and environmental risks.

The strong oxidizing properties of permanganic acid make it suitable for a wide range of applications. It is commonly used in organic synthesis, water treatment, and disinfection, as well as treatment of contaminated soil and groundwater [6]. It is also used in analytical chemistry to determine the concentration of reducible substances in a solution. In the context of Iron-Chromium Redox Flow Batteries (ICRFBs), permanganic acid is used to etch graphite felt electrodes, a surface treatment reported to reduce charge transfer resistance and support improved electrochemical performance [7]. In the capacitor design industry, the thermo-decomposition of highly concentrated permanganic acid is used to produce a solid, thin, continuous, and highly adherent manganese oxide film for high-performance capacitors [8]. Permanganic acid is also involved in the purification process of silicon and germanium semiconductors by eliminating carbon impurities through oxidation [9].

In the nuclear industry, permanganic acid has been recognized as an important agent for decontamination processes since 1978. It is used by industry professionals to remove oxide film deposited on internal structural metal components made of chromium, cobalt, and iron-based alloys, such as stainless steel, nickel-based alloys such as Inconel 600 and 690, and cobalt chromium alloys such as stellite, located in both the primary and secondary circuits of nuclear reactors [10,11,12]. This application is essential for preventive maintenance and extending the lifespan of key components like steam generators [13], and the safe dismantling of nuclear facilities, by minimizing radiation exposure and facilitating the classification and treatment of radioactive waste prior to final disposal [14,15].

Several approaches for the production of HMnO_4_ have been reported in the literature. Early studies described chemical oxidation of manganese compounds using strong oxidants, but these procedures were often limited in applicability [16,17]. More recently, electrochemical oxidation has emerged as a reliable method, involving the electrolysis of manganese precursors in acidic media. Although relatively costly, this route is recognized for providing HMnO_4_ solutions of high purity yield [18]. In addition, photoelectrochemical techniques have been explored. A study by Nakajima demonstrated an environmentally friendly production method based on a solar-driven photoelectrochemical process utilizing porous WO_3_ thin film photoanodes to generate permanganic acid [19].

Currently, the Ion Exchange Resin method has become the most favorable method for producing HMnO_4_ due to its operational simplicity and scalability for industrial application, and ability to produce high-yield and high-purity solution [20]. The ion exchange resin process involves passing a solution of KMnO_4_, the most stable form of permanganate, through a column or reactor filled with a strong acid cation exchange resin. During the process, the potassium ion is exchanged with a hydrogen ion from the resin (${R-SO}_{3}^{-}H^{+}$) resulting in the formation of permanganic acid according to the following cation exchange equilibrium reaction of species at the solid/liquid interface:

(1) $$R-{SO}_{3}^{-}H^{+}+{KMnO}_{4}⇌R-{SO}_{3}^{-}K^{+}{+HMnO}_{4}$$

According to the study by [21] for dilute aqueous solutions at room temperature and for different cations of the same charge, affinity for the sulfonate resin generally increases with the element’s atomic number: Li^+^ < H^+^ < Na^+^ < NH_4_^+^ < K^+^ < Cs^+^ < Ag^+^ < Tl^+^. The apparent exchange constant, therefore, depends on the proportion of the two cations in the equilibrium solution. For example, a sulfonic acid resin has a very high affinity for the alkali cation in solution, in the presence of large quantities of alkali in low acid concentration. Under these conditions, the reaction equilibrium shifts towards the formation of HMnO_4_. This acid, HMnO_4,_ tends to decompose into manganese dioxide (MnO_2_) while releasing ozone, a hazardous gas at high concentrations.

This work focuses on the synthesis of permanganic acid via cation exchange and investigates its reactivity in dissolving chromium oxide-an oxide phase found in passivation layers of metals. The performance of HMnO_4_ is evaluated in terms of its stability and efficiency, with the aim of optimizing its use in nuclear decontamination processes [22,23].

## 2. Materials and Methods

### 2.1. Permanganic Acid Preparation

HMnO_4_ solution was synthesized through a chromatographic resin exchange process using strong acid cation exchange AmberLite IRN97 H resin (Chauny, France), a nuclear grade with uniform spherical particle size. This resin contains a minimum of 99% exchange sites in the hydrogen form, indicating its high purity and efficiency for cation exchange applications. Total Exchange Capacity ≥ 2.10 eq/L (H^+^ form) and density = 820 g/L [24]. Potassium permanganate, KMnO_4_ (Sigma Aldrich, St Louis, MO, USA, 99% ACS Reagent), was used as the precursor for $MnO_{4}^{-}$ for ion exchange, where K^+^ is exchanged with the proton of the sulfonic group (Equation (1)). The synthesis is based on the exchange of H^+^ ions from the resin with K^+^ ions from KMnO_4_. Initially, potassium permanganate powder was dissolved in ultra-pure water and stirred for 24 h to ensure complete dissolution. Subsequently, the AmberLite IRN97 H resin was conditioned in a 190 ppm nitric acid solution 24 h prior to the experiment. The ion exchange process was initiated by introducing the KMnO_4_ solution into a column packed with the pre-conditioned resin. The eluate was collected, and the pH of both the inlet (KMnO_4_) prior to contact with the resin and the eluate was monitored. The pH difference serves as an indicator of the exchange and the degree of acidification in the resulting HMnO_4_ solution. To validate the ion exchange process, potassium concentrations in the collected fractions were measured using a Metrohm 850 Professional IC system (Villebon Courtaboeuf, France) equipped with a Metrosep C6 analytical column and a corresponding guard column. The eluent consisted of 504 ppm HNO_3_ and 82 ppm 2,6-pyridinedicarboxylic acid (PDCA). Prior to injection, samples were filtered through 0.45 µm PTFE membrane filters. If necessary, the samples were diluted with the eluent to fall within the calibration and pH range. Following the initial production trial, the preparation method was refined to optimize the purity and yield of the final HMnO_4_ solution.

### 2.2. Preliminary Evaluation of Ion Exchange Conditions

A series of three tests were carried out to evaluate the ion exchange performance of the resin, including its capacity and impact on solution pH (Table 1). Test 1 was performed under a reference stoichiometric condition (1:1 molar ratio between H^+^ resin sites and incoming K^+^). In Tests 2 and 3, distinct conditions were applied (higher KMnO_4_ concentration in Test 2, higher resin amount in Test 3) in order to evaluate the resin’s performance. In each test, the AmberLite IRN97 H resin was first preconditioned with a 190 ppm HNO_3_ solution, then packed into a column to interact with the KMnO_4_ solution. For every 5 mL of KMnO_4_ introduced, 1 mL aliquots were collected, and both pH and K^+^ ion concentration were measured.

- In Test 1, a low concentration of KMnO_4_ (316 ppm) was used with 0.49 g of AmberLite IRN97 H resin, calculated based on a 1:1 molar ratio between H^+^ and K^+^.
- In Test 2, the concentration of KMnO_4_ increased tenfold (3610) while keeping the resin quantity constant. This test aimed to provide insights into its maximum ion exchange capacity and observe any possible changes in pH or other chemical interactions that might occur under these conditions.
- In Test 3 the resin quantity increased to 1.48 g while maintaining the KMnO_4_ concentration at 316 ppm, as in Test 1.

### 2.3. Potassium Ion Monitoring by Ion Chromatography

Following the initial screening, further tests were carried out to refine the ion exchange process and assess its efficiency in more detail. KMnO_4_ solution was passed through the resin column, and fractions were collected every 2.5 mL, immediately as they emerged from the outlet of the column, without any additional waiting time between KMnO_4_ addition and collection. The potassium ion concentration in each fraction was determined using ion chromatography, providing a profile of exchange performance over the elution volume. This detailed analysis not only validated the exchange mechanism but also supported the fine-tuning of the production protocol. These optimized conditions enabled the production of a highly pure HMnO_4_ solution, with improved yield and minimal residual K^+^ content.

### 2.4. Cr_2_O_3_ Kinetic Dissolution in HMnO_4_ Solution

The dissolution kinetics of Cr_2_O_3_ in HMnO_4_ solutions were investigated at two concentrations: 240 ppm and 1920 ppm. For each experiment, 1 mg of Cr_2_O_3_ powder was added to 50 mL of HMnO_4_ solution in a glass vessel under a fume hood. The suspensions were maintained at constant temperatures of 30 °C, 40 °C, and 80 °C to assess the oxidation of Cr (III) into its soluble hexavalent form, Cr (VI). For each test, aliquots of 1–3 mL were collected at 1 min, 1 h, 3 h, 4 h, 24 h, 27 h, 31 h, and 33 h. The chromium concentration in each sample was determined by ICP-MS to monitor the evolution of dissolution over time. This experiment aimed to investigate the kinetics of the dissolution process and determine the influence of temperature and HMnO_4_ concentration on the efficiency of Cr_2_O_3_ oxidation. It is noteworthy that the experiments conducted at 80 °C were performed to reproduce the industrial conditions for decontamination processes involving the use of permanganate-based oxidants at elevated temperatures [25]. While these conditions are used to accelerate the reactions, they are not optimal for the stability of HMnO_4_.

### 2.5. Decomposing HMnO_4_ with Oxalic Acid

Following the dissolution of Cr_2_O_3_ by HMnO_4_, residual manganese species may remain in solution. Oxalic acid, which has a standard redox potential E^0^ (CO_2_/H_2_C_2_O_4_) = −0.49 V, is recognized for its ability to reduce the permanganate ion (${MnO}_{4}^{-}$) to manganese(II) ion (Mn^2+^) [26] according to the chemical reaction:

(2) $$5H_{2}C_{2}O_{4}+2MnO_{4}^{-}+6H^{+}⇌10CO_{2}↑+2{Mn}^{2+}+8H_{2}O$$

This reaction enables manganese to be removed from solution either by precipitation (e.g., Mn(OH)_2_ or MnCO_3_) or by co-precipitation with other insoluble phases (e.g., iron-based co-precipitation [27]. For each test, 50 mL of HMnO_4_ at concentrations of 240 ppm and 1920 ppm was mixed with 50 mL of oxalic acid (H_2_C_2_O_4_) at concentrations of 900 ppm, 3870 ppm, and 7560 ppm. The mixture was heated to 80 °C, a temperature close to those used in nuclear industry decontamination processes involving permanganic acid [28]. This is why, in the Cr_2_O_3_ powder experiments, the temperature was increased to 80 °C at high permanganic acid concentrations to better mimic real application conditions. The goal of decomposing HMnO_4_ with oxalic acid is to evaluate the effect of the H_2_C_2_O_4_/HMnO_4_ molar ratio on manganese reduction and removal under controlled thermal conditions.

### 2.6. Analytical Methods

Potassium and manganese (Mn^2+^) concentrations were determined by ion chromatography (IC) equipped with a conductivity detector. Prior to injection, samples were filtered through 0.22 µm membrane filters. The eluent consisted of a nitric acid (HNO_3_) and pyridine-2,6-dicarboxylic acid (PDCA) mixture, optimized for the separation of alkali metals. The system was calibrated using certified manganese and potassium standard solutions, and quantification was performed with a detection limit below 0.1 ppm.

Chromium concentrations in the dissolution experiments were measured using inductively coupled plasma mass spectrometry (ICP-MS) with a Thermo Scientific XSeries 2 instrument, (Thermo Scientific, Waltham, MA, USA). Samples were diluted in 2% ultrapure nitric acid and spiked with internal standards (Scandium-45 and Indium-115) to correct for signal drift and matrix effects. External calibration was performed using SCP Science solution standards, and the detection limit was approximately 4·10^−4^ mM.

## 3. Results and Discussion

### 3.1. Pre-Optimization of HMnO_4_ Production

#### 3.1.1. pH Evolution

**Figure 1:**
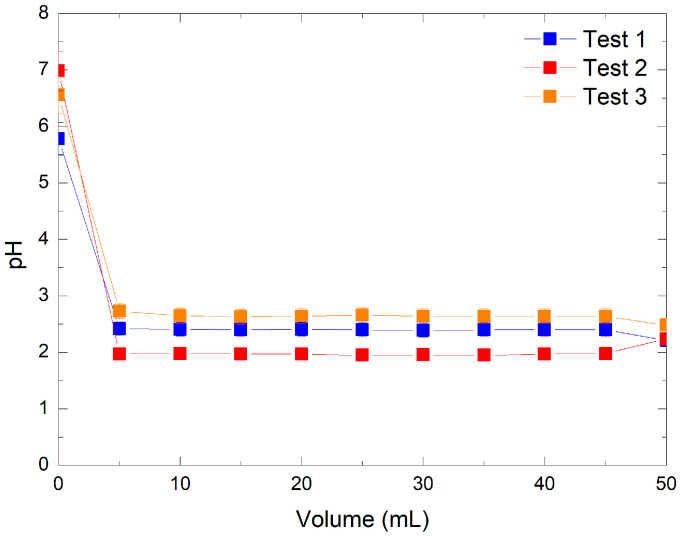
Comparative pH evolution in three preliminary optimization tests: Test 1 with 316 ppm KMnO_4_ and 0.49 g resin, Test 2 with 3160 ppm KMnO_4_ and 0.49 g resin, and Test 3 with 316 ppm KMnO_4_ and 1.48 g resin.

Three preliminary experiments were conducted on the acidification behavior of KMnO_4_ solutions following ion exchange with AmberLite IRN97 H resin. The pH of each 5 mL fraction was measured during the passage of KMnO_4_ solution through the resin column. The initial KMnO_4_ solution, referred as “volume 0”, had a pH between 5 and 7, depending on the concentration. As shown in Figure 1, increasing the concentration of KMnO_4_ led to a more significant decrease in pH upon contact with the resin.

The recovered solutions, identified as permanganic acid, exhibited strong acidity consistent with values reported by V. Subramanian and P. Chandramohan [20], where pH values ranged from 1.62 to 3.56. To further validate these observations, a comparison was made between experimental pH values and theoretical estimates based on the reported pKa of HMnO_4_ (between −4.5 and −2.25 [3,4,5]). A 316 ppm KMnO_4_ solution (Tests 1 and 3) corresponds to an expected pH of 2.70 for full conversion in HMnO_4_, while a 3160 ppm KMnO_4_ solution (Test 2) gives a pH of 1.70. These ranges are consistent with our experimental pH values in eluates with about 2.4 and 2.6 in Tests 1 and 3, respectively, and 1.9 in Test 2. The slightly lower values observed experimentally may be attributed to the residual nitric acid at 189 ppm from resin during the pre-conditioning. This comparison shows that most permanganate ions were effectively converted to HMnO_4_.

#### 3.1.2. K^+^ Ions Concentration

**Figure 2:**
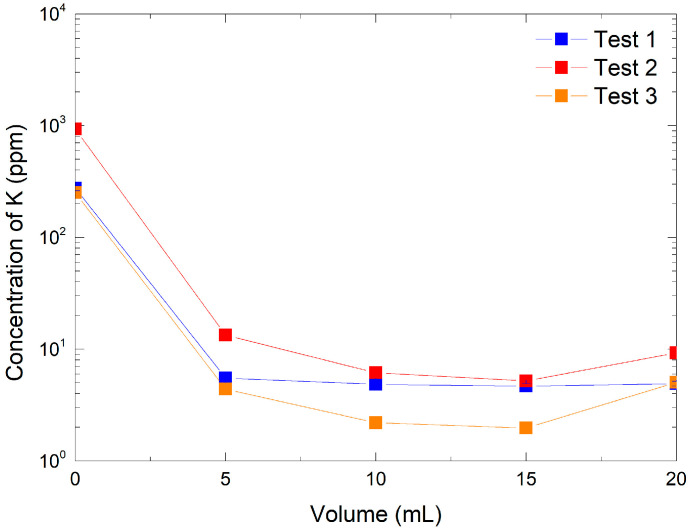
Potassium ion concentration analysis by ion chromatography in pre-optimization tests: Test 1 with 316 ppm KMnO_4_ and 0.49 g of resin, Test 2 with 3160 ppm KMnO_4_ and 0.49 g of resin, and Test 3 with 316 ppm KMnO_4_ and 1.48 g of resin.

To further validate the ion exchange process, potassium ion concentrations in each recovered fraction were measured. As shown in Figure 2, the resin demonstrated a high retention capacity for potassium at both tested KMnO_4_ concentrations. For the lowest concentration (316 ppm, Tests 1 and 3), K^+^ retention was nearly complete, with a measured efficiency of 99.95%, indicating that almost all potassium ions were replaced by protons from the resin during the exchange process. At the higher KMnO_4_ concentration (3160 ppm, Test 2), the retention efficiency slightly decreased to 97.9%, suggesting that the exchange capacity of the resin becomes a limiting factor when more potassium ions are introduced. This observation highlights the importance of optimizing the [K^+^]-to-resin ratio to maintain high exchange efficiency. These results confirm that the AmberLite IRN97 H resin is highly effective in exchanging K^+^ for H^+^ under the tested conditions. The minimal potassium content in the recovered HMnO_4_ solution further supports the successful production of high-purity acid, suitable for subsequent applications. However, we should note that potassium retention only corresponds to the efficiency of cation exchange. The concentration of MnO_4_^−^ was not directly evaluated during the experiments; therefore, we cannot completely exclude the possible limited decomposition of HMnO_4_ during or after elution.

### 3.2. Optimization of HMnO_4_ Production

**Figure 3:**
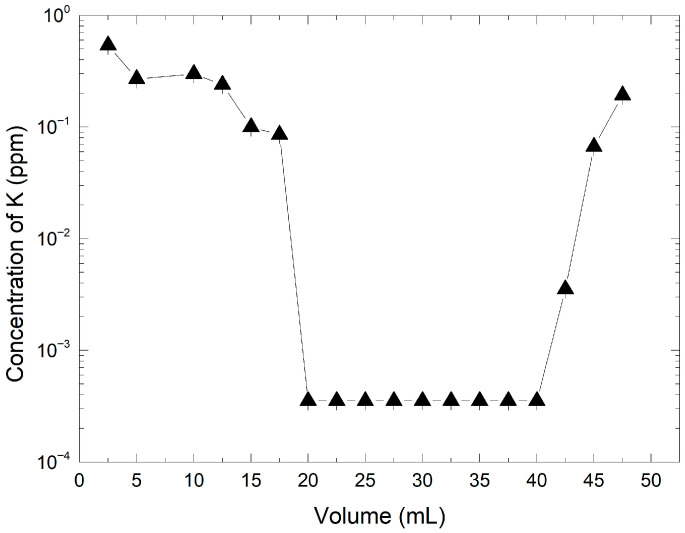
Concentration of K^+^ as a function of the collected volume of HMnO_4_ obtained during the ion exchange synthesis. The line with triangles indicates the potassium concentration determined in each fraction by ion chromatography.

Based on the results of the preliminary tests, an optimized protocol was implemented using 50 mL of a 3160 ppm KMnO_4_ solution and 1.48 g of resin. The eluate was collected in 2.5 mL fractions and analyzed for potassium ion content. As shown in Figure 3, the potassium concentration in the eluate was initially high but decreased progressively as the solution passed through the column. A stable low-concentration plateau was observed between 20 mL and 40 mL, with K^+^ concentrations below 0.1 ppm.

This plateau likely represents the effective exchange zone, where the majority of potassium ions were replaced by protons from the resin. Beyond this range, a gradual increase in K^+^ concentration was detected, suggesting a decrease in exchange efficiency due to partial saturation of the resin. The pH of the KMnO_4_ solution prior to exchange was 6.5 ± 0.1, while the recovered HMnO_4_ fractions reached a pH of approximately 1.63, consistent with successful conversion into the acid form. The K^+^ concentration in the initial KMnO_4_ solution was 255.6 ppm, whereas the maximum K^+^ concentration in the recovered solution is only 0.54 ppm, indicating a retention rate of 99.8%. The experimental results confirm that under optimized conditions, the AmberLite IRN97 H resin provides near-complete ion exchange, yielding a high-purity permanganic acid solution. The clearly defined exchange plateau also offers a practical window for collecting HMnO_4_ with minimal potassium contamination.

The observed potassium concentrations at the end of the experiment indicate resin exchange capacity saturation after eluting 42.5 mL of the KMnO_4_ solution. These observations are consistent with the results from pre-optimization Tests 1, 2, and 3 and demonstrate that the initial assumptions and the protocol applied for optimization are valid.

### 3.3. Evolution of Cr_2_O_3_ Dissolution in Presence of HMnO_4_ Solution

The oxidative dissolution of Cr_2_O_3_ by HMnO_4_ was investigated in order to assess its efficiency for removing passive oxide films. The overall process can be represented by the following reaction in acidic media to favor the reaction:

(3) $$5{Cr}_{2}O_{3}+6{HMnO}_{4}+2H^{+}\to 5{Cr}_{2}O_{7}^{2-}+6{Mn}^{2+}+4H_{2}O$$

Several concentrations of HMnO_4_ were investigated.

#### 3.3.1. At 240 ppm HMnO_4_

**Figure 4:**
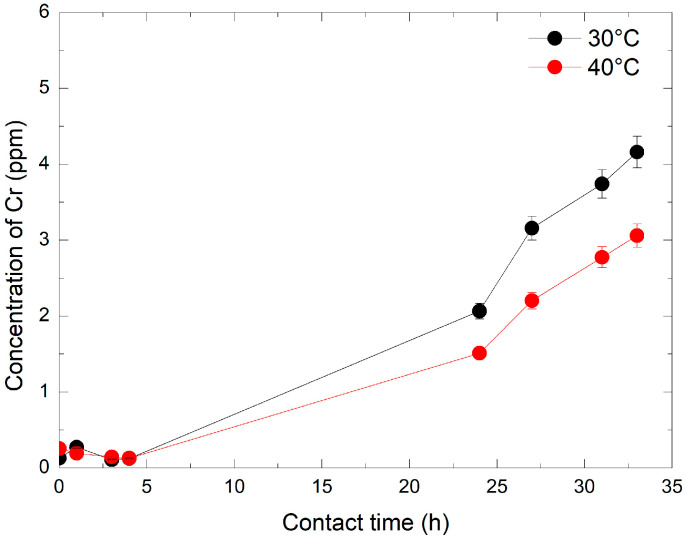
Evolution of Cr concentration by ICP-MS over time and temperature at 240 ppm of HMnO_4_.

The dissolution kinetics of Cr_2_O_3_ were first evaluated using a 240 ppm HMnO_4_ solution at 30 °C and 40 °C. Chromium concentrations were monitored by ICP-MS at specific intervals: immediately (1–2 min) and after 1 h, 3 h, 4 h, 24 h, 27 h, 31 h, and 33 h. As illustrated in Figure 4, Cr concentration remained stable at about 0.25 ppm during the first 4 h. A significant increase was observed after 24 h, with final concentration values rising by 6 and 8-fold at 40 °C and 30 °C, respectively. This trend continued over time, reaching final concentrations of 3 and 4.2 ppm at 40 °C and 30 °C, respectively, after 33 h. The results indicate that the dissolution of Cr_2_O_3_ is both temperature and time-dependent: the lower the temperature, the greater the ability of HMnO_4_ to dissolve Cr_2_O_3._ This is likely due to the increased instability of HMnO_4_ at increased temperatures. Furthermore, at a given temperature, the efficiency of HMnO_4_ in dissolving Cr_2_O_3_ increased proportionally with contact time.

#### 3.3.2. At 1920 ppm HMnO_4_

**Figure 5:**
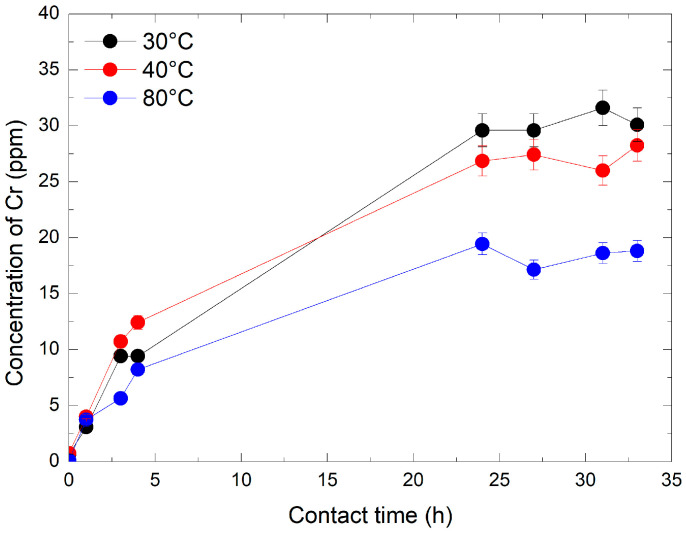
Evolution of Cr concentration by ICP-MS over time and temperature at 1920 ppm of HMnO_4_.

In the second dissolution experiment, Cr_2_O_3_ powder was dissolved in the presence of 1920 ppm HMnO_4_ at three different temperatures: 30 °C, 40 °C, and 80 °C. The chromium concentrations were measured at the following times: immediately (1–2 min), 1 h, 3 h, 4 h, 24 h, 27 h, 31 h, and 33 h. Figure 5 shows a significant increase in Cr concentrations within the first hour, especially at 30 °C and 40 °C. After 24 h, a plateau was observed. The experiment also revealed that at 80 °C, the amount of dissolved Cr was lower at 30 °C and 40 °C. These results highlight the influence of HMnO_4_ concentration on the dissolution of Cr_2_O_3_. After 4 h of reaction, the amount of dissolved chromium was significantly higher at 1920 ppm HMnO_4_, increasing by factors of 40 and 5 compared to the experiments with 240 ppm. An inverse relationship was observed between temperature and dissolved chromium concentration, with higher temperatures leading to reduced Cr dissolution. This trend is best explained by the thermal instability of permanganic acid (HMnO_4_), which decomposes more rapidly at elevated temperatures, reducing its oxidative effectiveness. These observations are consistent with previous findings [29], who reported increased HMnO_4_ decomposition with rising temperature at concentrations of 360 ppm and temperatures up to 95 °C. The highest levels of Cr dissolution were achieved at lower temperatures, highlighting the importance of HMnO_4_ stability and extended contact time for efficient Cr_2_O_3_ dissolution.

To assess the dissolution efficiency, the amount of Cr released into solution was compared to the theoretical chromium content of the initial Cr_2_O_3_ sample. Under the most favorable condition (30 °C, 1920 ppm HMnO_4_), approximately 31% of the total chromium was recovered in solution after 33 h. This partial but significant dissolution highlights the suitability of the experimental setup for monitoring the progressive oxidation of Cr (III) by HMnO_4_.

The results here also highlight the almost linear increase in Cr dissolution with increasing HMnO_4_ concentrations at the same contact times (about 3.5 ppm at 33 h vs. closer to 30 ppm at increased conc of HMnO_4_ at similar temps). This is interesting because this leads to a choice during the treatment or decontamination. A significantly lower contact time is attainable at elevated concentrations which has its benefits vs. a higher contact time at lower concentrations. This would require further exploration, considering factors such as resin and other costs, which are out of the scope of this work.

### 3.4. Effect of Oxalic Acid Concentration on HMnO_4_ Decomposition at 80 °C

**Figure 6:**
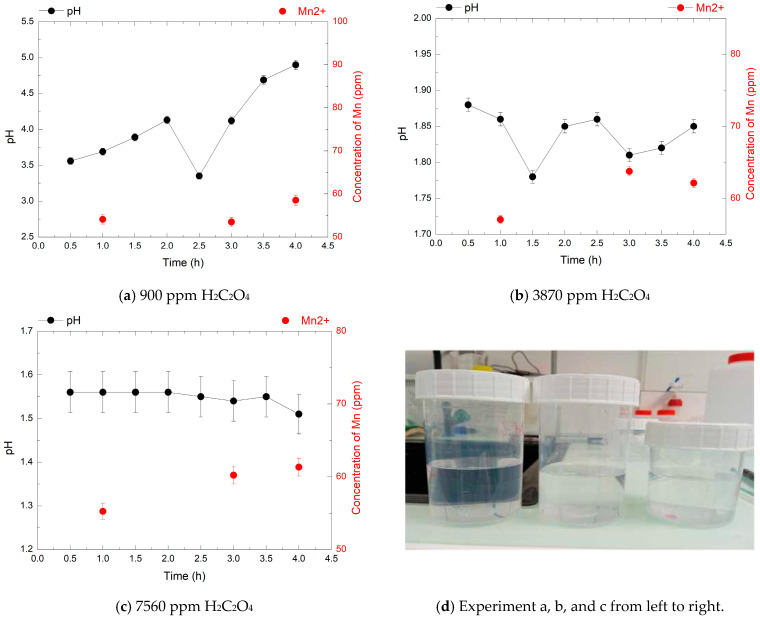
pH evolution (black points) and Mn^2+^ concentrations (red points) over 4 h during the decomposition of 240 ppm HMnO_4_ at 80 °C, in the presence of 900, 3870, and 7560 ppm of H_2_C_2_O_4_. Each Mn^2+^ point corresponds to a sampling time; pH was measured continuously.

**Figure 7:**
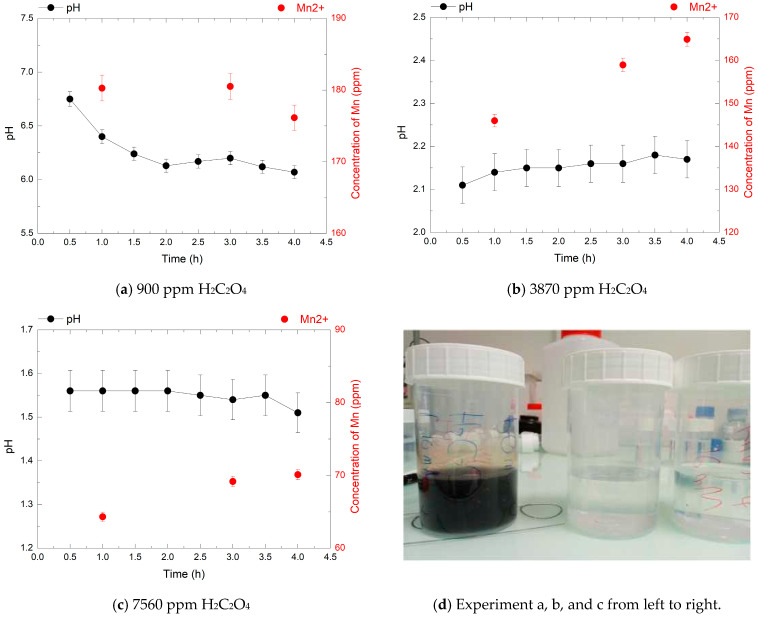
pH evolution (black points) and Mn^2+^ concentrations (red points) over 4 h during the decomposition of 720 ppm HMnO_4_ at 80 °C, in the presence of 900, 3870, and 7560 ppm of H_2_C_2_O_4_.

**Figure 8:**
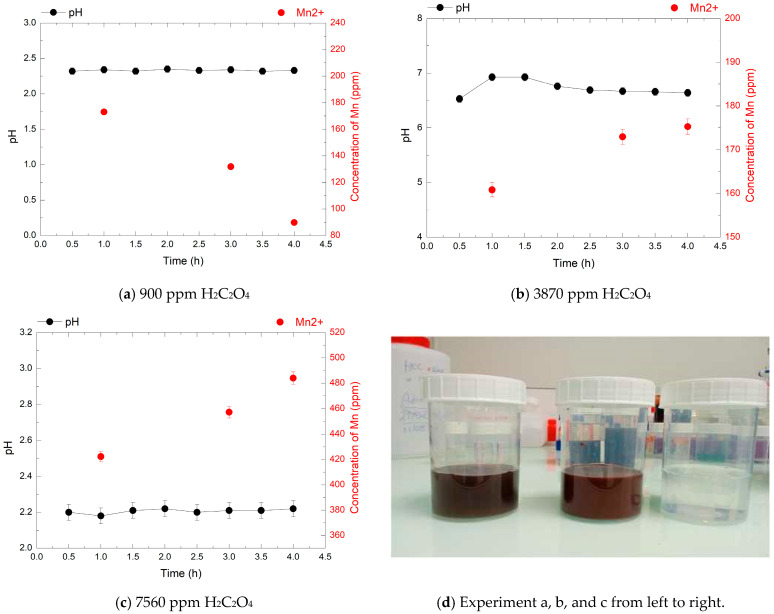
pH evolution (black points) and Mn^2+^ concentrations (red points) over 4 h during the decomposition of 1920 ppm HMnO_4_ at 80 °C, in the presence of 900, 3870, and 7560 ppm of H_2_C_2_O_4_.

The decomposition of HMnO_4_ by oxalic acid (H_2_C_2_O_4_) was investigated to determine the conditions required for complete reduction of permanganate species without manganese dioxide precipitation (Figure 6, Figure 7 and Figure 8). HMnO_4_ solutions at 240, 720, and 1920 ppm were reacted with increasing concentrations of H_2_C_2_O_4_ (900, 3870, and 7560 ppm) at 80 °C. The evolution of Mn^2+^ concentration, pH, and the presence of MnO_2_ precipitation were monitored over 4 h to assess the effect of the H_2_C_2_O_4_/HMnO_4_ molar ratio on the reduction efficiency. This step is very crucial because in nuclear decontamination processes, any residual permanganate from the decontamination step remains as a byproduct and must be treated as waste. Efficient decomposition of this residual permanganate into manganese precipitate (MnO_2_), which can be separated, is essential to minimize secondary liquid waste generation and ensure proper waste management.

In the first experiment, with 240 ppm HMnO_4_, the Mn^2+^ concentration remained nearly constant (about 60 ppm) across all oxalic acid concentrations, corresponding to ~55% of the theoretical yield expected for full reduction.

The pH, monitored every 30 min, increases from around 3.5 to 5 for the 900 ppm concentration, and tends to become more acidic for the 3870 ppm and 7560 ppm concentrations and no visible MnO_2_ precipitation was observed, suggesting a predominantly homogeneous reduction. Lower concentrations of oxalic acid led to higher final pH values, with a maximum of pH 5 at 900 ppm H_2_C_2_O_4_. The pH minimum at 2.5 h likely reflects a transient stage in the redox reaction, where a proton release causes a temporary drop—indicative of a complex reaction mechanism.

These results suggest that oxalic acid can effectively reduce HMnO_4_ without inducing manganese precipitation, particularly at moderate concentrations and elevated temperature (80 °C). However, the observed reduction efficiency, especially at lower HMnO_4_ concentrations (240 ppm), remained incomplete despite increasing oxalic acid doses. This behavior may partly stem from oxalic acid’s thermal degradation, which limits its reducing capacity over time. A related study showed that heating oxalic acid in nitric acid at 100 °C in the presence of Mn^2+^ significantly accelerates its decomposition [30].

In the second experiment, with 720 ppm HMnO_4_, the final Mn^2+^ concentrations after 4 h were 176 ppm, 165 ppm, and 70 ppm for 900, 3870, and 7560 ppm oxalic acid concentration, respectively. These correspond to 53%, 50%, and 21% of the theoretical value. At low oxalic acid concentration (900 ppm), MnO_2_ precipitation was visually observed, likely due to the combination of low reductant availability and a rising pH (up to 6.1), indicating an incomplete reduction reaction.

In this experiment, with 3870 ppm HMnO_4_, the reaction proceeded more efficiently, with a stable acidic pH (pH 2.15), the absence of visible precipitate, and a high Mn^2+^ concentration consistent with reduction. At 7560 ppm H_2_C_2_O_4_, the pH remained low (1.55), and no precipitation was observed, yet the Mn^2+^ concentration decreased significantly. This suggests that other reactions may compete, potentially leading to the formation of soluble or colloidal intermediate species. Further investigation would be needed to clarify the fate of manganese under these conditions.

In the third experiment, with 1920 ppm HMnO_4_, the Mn^2+^ concentrations measured after 4 h were 90 ppm, 175 ppm, and 485 ppm for 900, 3870, and 7560 ppm oxalic acid, respectively. These correspond to 10%, 20%, and 55% of the theoretical yield, indicating that only partial reduction was achieved in all cases. At 900 ppm H_2_C_2_O_4_, the limited availability of reductant resulted in minimal Mn^2+^ concentration despite a moderately acidic pH (2.3). At 3870 ppm the Mn^2+^ concentration increased but the pH stabilized between 6.1 and 6.8, suggesting possible proton depletion that may have limited reduction efficiency. The highest Mn^2+^ concentration was obtained at 7560 ppm oxalic acid, under acidic conditions (pH 2.2), with no visible MnO_2_ precipitation. This condition resulted in the most efficient reduction observed at this HMnO_4_ concentration, approaching 55% of the expected value. However, with lower concentrations, complete reduction was not achieved, likely due to the high oxidant load and precipitation reactions competing with direct Mn^2+^ formation.

The reduction of HMnO_4_ by oxalic acid appears to be influenced by several interdependent factors. While maintaining a low pH is essential to promote the reduction of Mn (VII) to Mn (II), this condition alone does not ensure complete reduction. In all experiments, the Mn^2+^ yield remained systematically below the theoretical value, not exceeding 55%, even under strongly acidic conditions and in the presence of a large excess of reductant. This limitation suggests that other processes are competing with or limiting the full reduction. In particular, the reaction may be sensitive to temperature: at 80 °C, oxalic acid can decompose, as observed during the thermal stability experiments of HMnO_4_ in the presence of Cr_2_O_3_ powders. This could contribute to the incomplete reaction observed, even in the absence of visible manganese oxide precipitation.

Furthermore, the formation of colloidal or complex manganese species that remain in solution but are not detected by ion chromatography may also contribute to the limited Mn^2+^ yield. Similar behavior was reported by H.-C. Eun and S. Yoon Park [31], who showed that an excess of reductant (N_2_H_4_ in their work) in acidic media can lead to the formation of insoluble manganese oxides or intermediate species, thereby limiting Mn^2+^ recovery. The observed sensitivity to the H_2_C_2_O_4_/HMnO_4_ molar ratio further confirms that the reaction is affected not only by the proton concentration but also by the relative amount of available reductant.

The overall results indicate that HMnO_4_ reduction by oxalic acid is influenced by multiple parameters, including reductant concentration, pH, and temperature. A significant contributing factor is likely the thermal decomposition of oxalic acid at 80 °C, which can reduce its effective concentration over time. Prior studies have shown that oxalic acid degrades more rapidly at elevated temperatures, especially in oxidative media. For instance, complete decomposition of 47,000 ppm oxalic acid was observed within 4 h at 100 °C in nitric acid with 2570 ppm Mn^2+^, whereas it required over 30 h without the catalyst [30]. This reinforces the notion that oxalic acid instability under thermal and oxidative stress may limit its reductive capacity in HMnO_4_ systems.

## 4. Conclusions

This study explored the synthesis of permanganic acid HMnO_4_ by cation exchange using a nuclear-grade Amberlite IRN97 H resin and its application for the oxidative dissolution of chromium oxide. The method produced a highly acidic solution of HMnO_4_ with low potassium content, confirming the effectiveness of the resin-based process. Potassium removal reached 99.95%, and the resulting solution exhibited a pH range between 1.65 and 2.5.

The results highlight the influence of both HMnO_4_ concentration and temperature on the efficiency of Cr_2_O_3_ dissolution. Higher acid concentrations favored reactivity over time, while lower temperatures improved HMnO_4_ stability. This study allowed us to refine the synthesis of HMnO_4_, a relevant oxidant for the chemical dissolution of chromium-based alloys, particularly in the nuclear industry.

The decomposition of HMnO_4_ by oxalic acid was also investigated to evaluate the fate of manganese after the oxidative step. The reduction in efficiency depended not only on the acidity of the medium, but also on the H_2_C_2_O_2_/HMnO_4_ molar ratio. A minimum ratio above 2.75 appeared necessary, but the best results were obtained between 5 and 7, depending on the initial concentration of HMnO_4_.

It is important to note that the release of ozone during HMnO_4_ decomposition could pose significant safety challenges. Although concentrations used in the lab remain below harmful thresholds, scale-up for industrial decontamination must account for gas handling and mitigation. Extending experiments to include temperatures below and above 80 °C (e.g., 25–95 °C) would help pinpoint thermal stability thresholds of both HMnO_4_ and oxalic acid more precisely. Sampling Mn^2+^ and pH at shorter intervals, especially within the first hour, would provide better kinetic insights into decomposition and reduction steps and help model the reaction progression.

These results are relevant to advanced nuclear decontamination processes such as HP-CORD (Chemical Oxidation and Reduction Decontamination) [27], where permanganic acid is used to dissolve passivation layers rich in chromium oxide. The moderate HMnO_4_ concentrations used in this study (up to 1920 ppm) align with operational concentrations in actual decontamination systems.

## Figures and Tables

**Table 1:** Quantities of materials used in pre-optimization tests.

	KMnO_4_ (ppm)	Resin (Grams)	HNO_3_ (ppm)
Test 1	316	0.49	190
Test 2	3160	0.49	190
Test 3	316	1.48	190

## Data Availability

The data supporting the findings of this study are available from the corresponding author upon request.
